# From Incivility to Turnover Intentions among Nurses: A Multifoci and Self-Determination Perspective

**DOI:** 10.1155/2023/7649047

**Published:** 2023-07-21

**Authors:** Lixin Jiang, Xiaohong (Violet) Xu, Stephen Jacobs

**Affiliations:** ^1^University of Auckland, Auckland, New Zealand; ^2^Department of Management, The University of Texas at San Antonio, San Antonio, TX 78249, USA

## Abstract

**Purpose:**

We investigate the associations between incivility from multiple sources (i.e., doctors, supervisors, fellow nurses, and patients/visitors) and nurse turnover intentions. We take a self-determination perspective to explore whether psychological needs for autonomy, belongingness, and competence explain the relationship between incivility and turnover intentions. Finally, we examine whether incivility from doctors, supervisors, fellow nurses, and patients/visitors may primarily relate to one of the three basic psychological needs and whether the autonomy need may have the strongest relationship with turnover intentions.

**Design:**

A three-wave time-lagged design was used. Each measurement point was separated by one workweek. New Zealand nurses were asked to evaluate their workplace incivility at Time 1, three basic psychological needs at Time 2, and turnover intentions at Time 3. *Findings*. Supervisor incivility directly related to turnover intentions. The autonomy need was the only significant mechanism underlying the relationships of incivility from doctors, supervisors, and fellow nurses with turnover intentions. In addition, doctor incivility related to the autonomy need, patient/visitor incivility was not significantly related to any psychological needs, and incivility from fellow nurses and supervisors related to psychological needs for belongingness and autonomy. *Originality*. This study takes a multifoci perspective to examine nurse incivility from multiple sources. The novelty lies in the introduction of self-determination theory to the understanding of workplace incivility. Finally, we turn the spotlight on the importance of examining whether incivility from different sources may be primarily related to different psychological needs and whether the autonomy need plays a key role in turnover intentions.

## 1. Introduction

Nurse turnover is a growing problem currently facing the health care sector worldwide [1]. Duffield et al. [2] reveals that New Zealand has an alarmingly high rate of nurse turnover at 44.3% [3], significantly higher than that of the U.S. (26.8%), and more than double that of Canada (19.9%) and Australia (15.1%). More recently, a New Zealand Nurses Organization survey [4] reveals that many nurse leaders had difficulty in retaining existing nurses or recruiting new ones. Not surprisingly, nurse turnover costs organizations significantly. For example, it can cost up to $23,800 per registered nurse turnover in New Zealand, which is about half of an annual registered nurse salary [3]. Nurse turnover also puts extra workload on fellow nurses and compromises patient care [5]. Given that turnover intentions represent one of the strongest predictors of actual turnover [6], this study aims to identify predictors of nurse turnover intentions.

The existing literature has summarized many antecedents of nurse turnover intentions, including organizational factors (e.g., organizational ethical climate), work-related factors (e.g., work overload), and employee factors (e.g., years of experience [7]). Building on this line of research, the recent literature has begun to explore how workplace incivility may contribute to nurse turnover intentions [8].

Workplace incivility can be defined as “low-intensity deviant behavior with ambiguous intent to harm the target, in violation of workplace norms for mutual respect. Uncivil behaviors are characteristically rude and discourteous, displaying a lack of regard for others” ([9] p. 457). Unfortunately, the number of nurses who are victims of workplace incivility is staggeringly high. For example, Lewis and Malecha [10] reveal the prevalence rate of incivility among nurses over the previous year as 84.8%. Indeed, studies from various countries [11] have documented high levels of uncivil experiences among nurses. New Zealand is no exception. A recent survey documents that 63% of 1,436 nurse respondents experienced verbal abuse from patients/visitors in the previous year [12].

Given the prevalence of nurse experienced incivility, we focus on the relationship between workplace incivility and nurse turnover intentions. According to self-determination theory (SDT [13]), when the work environment frustrates people's needs for autonomy, belongingness, and competence, individual functioning suffers. Specifically, the need for autonomy represents a sense of volition, control, and choice over one's actions. The need for belongingness is a sense of connection or the need to belong to a larger group. Finally, the need for competence refers to a sense of self-efficacy and perceived ability to obtain desired outcomes.

Workplace incivility may frustrate these three basic human needs, which in turn, relate to subsequent turnover intentions. Specifically, incivility forces nurses into a situation that they do not initiate nor desire. As the targets of incivility, nurses have few possibilities to change the situation or prevent future incivility. Therefore, workplace incivility may affect nurses' sense of choice and volition, frustrating their need for autonomy [14]. Moreover, incivility deprives nurses of a sense of belonging with others. When individuals are “treated like air” or made to suffer the “silent treatment” at work [15], this may affect nurses' sense of communion and threaten their need to belong. An individual's inability to effectively deal with other difficulties in the workplace can affect their sense of mastery over their environment and their ability to achieve desired outcomes. Thus, being exposed to workplace incivility may threaten one's sense of being a capable individual [14]. On the other hand, frustrating the need for autonomy, belongingness, and competence has been found to relate to turnover intentions [16]. Together, it suggests that the needs for autonomy, belongingness, and competence may partially explain the relationship between incivility and turnover intentions.

Moreover, SDT suggests that each psychological need may *independently* explain the relationships between features of the work environment and outcomes [16]. However, the vast majority of studies on workplace incivility tend to lump incivility from different sources together, failing to differentiate the potentially different influences of each source [17]. Consequently, we have limited knowledge about whether incivility from different sources (e.g., supervisor, coworker, and customer) may have different relationships with outcomes of interest. However, given role differentials, nurses may react to uncivil behaviors from different sources via different mechanisms [18], that is, top-down (incivility from doctors and direct supervisors), lateral (incivility from nurse coworkers), and outside incivility (incivility from patients/visitors [19, 20]) may have differential relationships with psychological needs (cf. [18]). Specifically, because doctors have high status according to the hierarchy within the health care systems and nurse supervisors are in direct control of rewards and punishment, both doctors and supervisors are in a position of power. Thus, nurses' need for autonomy may be frustrated when experiencing incivility from doctors and/or supervisors who have formal power over the target and have control over important resources (e.g., rewards and promotion) in the hospital setting, that is, when incivility comes from those in positions of power, it can create barriers for nurses, limit their choices and initiatives, and deny them a sense of volition and psychological freedom. Thus, top-down incivility can lead to frustration of the nurses' need for autonomy.

The need for belongingness is a fundamental human drive [21]. This need to be accepted, cared for, and loved by members of a group is especially salient when the group consists of those who are similar to oneself in key aspects (e.g., an ingroup), such as the coworker group [18]. For example, exclusion by an ingroup feels worse than exclusion by an outgroup, while inclusion from an ingroup is more fulfilling to one's belongingness need than inclusion from an outgroup member [22]. Thus, when fellow nurses display uncivil behaviors, it communicates to the target that they are not a well-respected member of the nursing group and do not belong, thereby especially thwarting their need for belongingness.

Finally, the need for competence is especially undermined when patients and visitors provide negative feedback (cf. [23]) via uncivil behaviors towards nurses, distrust information given by nurses, question nurses' abilities, and doubt their achievements. When patients and/or visitors show disrespect and act rudely with nurses, signaling ineffectiveness [24], nurses may feel that their work is ineffective and difficult and doubt their own abilities, thereby threatening their need for competence.

Finally, because each need represents an independent construct [23] and explains unique variance in outcomes of interest [16], the three psychological needs for autonomy, belongingness, and competence may have differential relationships with turnover intentions. Of the three needs for autonomy, belongingness, and competence, satisfying the need for autonomy by allowing nurses to volitionally carry out desired activities is the most crucial for experiencing intrinsic motivation, that is, fulfilling the need for autonomy enables nurses to grasp the importance of organizational values, fully integrate them, and transform organizational values and regulations into their own; thus, strongly relating to decreased turnover intentions. Together, we predict that

  Hypothesis 1: the relationship between exposure to incivility, including doctor incivility (H1a), supervisor incivility (H1b), coworker incivility (H1c), and patient/visitor incivility (H1d), and turnover intentions is partially mediated by psychological needs for autonomy, belongingness, and competence
  Hypotheses 2–4: sources of workplace incivility differentially predict psychological needs for autonomy, belongingness, and competence, that is, incivility from doctors (H2a) and supervisors (H2b) has the strongest relationship with the autonomy need, coworker incivility has the strongest relationship with the belongingness need (H3), and patient/visitor incivility has the strongest relationship with the competence need (H4)
  Hypothesis 5: among the three basic psychological needs, the autonomy need has the strongest relationship with turnover intentions

**Figure 1:**
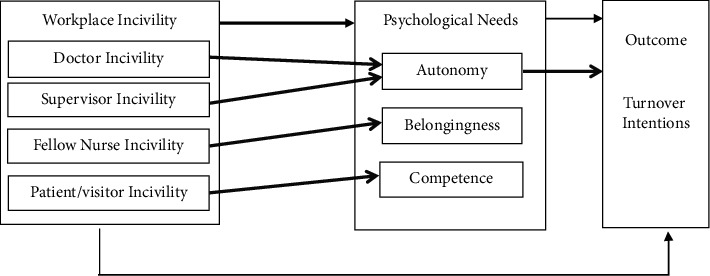
Hypothesized relationships among workplace incivility, psychological needs, and turnover intentions. Note. Lines in black indicate a partial mediation model from workplace incivility to turnover intentions via three psychological needs. Lines in grey indicate that each source of workplace incivility is primarily related to one of the psychological needs (e.g., fellow nurse incivility primarily frustrates the need for belonging), and the autonomy need is primarily related to turnover intentions.

Together, we take a multifoci approach [17] and adopt SDT [13] to understand why and how incivility from different sources (i.e., doctors, supervisor, fellow nurses, and patients/visitors) may be related to nurse turnover intentions differently in a sample of NZ nurses (see Figure 1). By providing a comprehensive and fine-grained examination of nurse-experienced incivility, we extend past research on nurse incivility, that was based on stress-related (e.g., Conservation of Resources Theory in [25]; Job Demands-Resources Model in [26]; Affective Event Theory in [27]) and social exchange (e.g., [28]) related theoretical frameworks.

## 2. Methods

### 2.1. Participant and Procedures

We used a three-wave time-lagged design to examine our hypotheses. Power analysis for structural equation modelling (SEM) requires extensive priori information about the intercorrelations among variables as well as the exact patterns (e.g., effect sizes) of the paths. As a result, it is often challenging and unfeasible to conduct power analysis for SEM. Thus, the sample size calculation in SEM is typically determined based on the number of parameters. A widely accepted rule of thumb is 5 : 1 ratio of cases to free parameters [29] or more strictly 10 : 1 ratio of cases to free parameters [30]. In our SEM model, there were eight variables (i.e., four sources of incivility, three psychological needs, and one outcome), resulting in 30 free parameters in the path analysis model. Therefore, the corresponding sample size would be 150 based on the 5 : 1 ratio of cases to free parameters [29], or 300 based on the 10 : 1 ratio of cases to free parameters [30]. Following the strict standard, we aimed to have about 300 participants at Time 3. Because we expected to have 50% of attrition rates over time, we aimed to have over 600 nurses register their interest during the recruitment stage.

The study was approved by the human participants' Ethics Committee of the first author's university (reference number: 021691). Upon receiving ethical approval in July 2018, we started to recruit participants. We used several avenues to recruit New Zealand nurses where we approached the Directors of Nursing from three District Health Boards of New Zealand, posted the advertisements of this study on the bulletin boards in New Zealand hospitals, and had the NZNO post our study on their Facebook page and newsletters. NZNO is the leading professional body of nurses and nursing union in New Zealand, representing more than 55,000 nurses and health care workers.

If the nurses were interested in participating, they were directed to an anonymous recruitment survey where we asked them to provide their email address, confirm their eligibility for participation, and enter the end date of their current (or future) workweek shift, given that nurses did not work the common Monday–Friday weekly shift. To be eligible for participation, participants must be over 18 years, registered nurses, and have worked for the current organization more than six months. The first eligible participant who entered their email address was on 28th Sept 2018, while the last one was 30th Jan 2019.

Based on the end date of their workweek shift, we then sent participants survey invitations via their email addresses, which allowed us to match each survey across time. Because nurses had their own working schedules, we sent out each weekly survey at different time points to accommodate each nurse's schedule. The first survey to the first group of nurses was sent on 30th Sept 2018, while the last survey to the last group of nurses was sent on 15th Feb 2019. Notably, for the same nurse, each survey was separated by one workweek shift. For example, the first group of nurses completed their surveys on 30th Sept 2018, 4th Oct 2018, and 7th Oct 2018, whereas the last group of nurses completed their surveys on 12th Feb 2019, 18th Feb 2019, and 23rd Feb 2019.

We followed the same cohort of nurses across time. Surveys were hosted on Qualtrics. Workplace incivility was assessed at Time 1, three basic psychological needs at Time 2, and turnover intentions at Time 3.

### 2.2. Measures

#### 2.2.1. Workplace Incivility

The Nursing Incivility Scale (NIS; [31]) was used to measure incivility from multiple sources. Specifically, NIS evaluated nurses' experiences of incivility from four sources, including doctors, direct supervisors, coworkers, and patients/visitors. Incivility from doctors was evaluated by seven items; a sample item was “Doctors are condescending to me;” the reliability of this dimension in our study was 0.89. Incivility from direct supervisors was assessed by seven items; a sample item was “My direct supervisor does not respond to my concerns in a timely manner;” the reliability of this dimension in our study was 0.87. Incivility from coworkers was evaluated by seven items; a sample item was “Other nurses on my unit claim credit for my work;” the reliability of this dimension in our study was 0.90. Finally, incivility from patients/visitors was evaluated by five items; a sample item was “Patients/visitors do not trust the information I give them and ask to speak with someone of higher authority;” the reliability of this dimension in our study was 0.87. These items were rated on a 5-point scale ranging from 1 (*Never*) to 5 (*A great deal*).

#### 2.2.2. Basic Psychological Needs

The need satisfaction scale [32] was used to evaluate nurse basic psychological needs at work, including satisfaction of the belongingness need, the competence need, and the autonomy need. Specifically, satisfaction of the belongingness need was assessed by six items; a sample item was “At work, I feel part of a group;” the reliability of this dimension in this study was 0.85. Satisfaction of the competence need was measured by four items; a sample item was “I really master my tasks at my job;” the reliability of this dimension in this study was 0.89. Satisfaction of the autonomy need was evaluated by six items; a sample item was “I feel like I can be myself at my job; ” the reliability of this dimension in this study was 0.81. These items were assessed on a 7-point scale ranging from 1 (*strongly disagree*) to 7 (*strongly agree*).

#### 2.2.3. Turnover Intentions

Three items developed by Hanisch and Hulin [33] were used to measure turnover intentions (*α* = 0.94). A sample item was “I have thought about leaving this job.” The scale was rated on a 5-point scale ranging from 1 (*Never*) to 5 (*A great deal*).

### 2.3. Control Variables

We controlled for age, organizational tenure, gender, and permanent versus temporary employment status in our analyses.

### 2.4. Data Analysis

Path analysis was used to test our hypotheses with Mplus 8.4 with full information maximum likelihood (FIML) dealing with missing data [34]. We used FIML because scholars have recommended FIML to deal with missing data for more accurate estimates of standard errors and higher statistical power, resulting in more accurate hypothesis tests (e.g., [35, 36]).

We evaluated the models based on various fit indices, including the comparative fit index (CFI [37]), root mean square error of approximation (RMSEA [38]), and the standardized root mean square residual (SRMR [39]). Ideally, the model with adequate fit should be with CFI greater than 0.95, RMSEA less than 0.06, and SRMR less than 0.08 [39]. There has been much debate on the use of the goodness-of-fit indices, such as the cutoff values, to accept or reject a model. Although Hu and Bentler [39] provided general guidelines for assessing goodness-of-fit indices, they and other statisticians (e.g., [40, 41]) have cautioned against indiscriminately relying on these cutoff criteria. In addition, scholars have cautioned that it is not appropriate to use one single fit index to reject or accept a model [42]. Indeed, research suggests that fit indices can demonstrate substantial variability across different data conditions, indicating that the cutoff values may be too strict or too lenient in some cases, and can be biased in certain situations. Different fit indices have varying sensitivity to external factors such as sample sizes and different statistical techniques, leading to increased variability across fit indices (e.g., [43–45]). For instance, some fit indices, such as CFI and RMSEA, are less subject to the impact of extraneous variables [46]. Because RMSEA can be determined by multiple factors, such as the complexity of the model and the sample size [47], researchers do not recommend a “fixed target” value for RMSEA [48, 49]. Given the debate on the criteria for assessing goodness-of-fit indices and substantial variation of each fit index across different conditions, it is advisable to view each fit index as merely suggesting a specific aspect of the model fit. Therefore, instead of relying solely on a single fit index, we evaluated our model using multiple fit indices, following the guidelines provided by Hu and Bentler [39], which are widely recognized as the standard criteria for assessing goodness-of-fit.

## 3. Results

### 3.1. Descriptive Statistics and Robustness Checks

In total, there were 674 qualified nurses who registered their interest. At time 1, we received valid responses from 413 eligible nurses; at Time 2, we contacted these 413 nurses and received valid responses from 339 nurses; and at Time 3, we contacted those 339 nurses and received valid responses from 294 nurses. All participants who provided valid responses at Time 1 (*N* = 413) were included in the analyses. Most of the sample were female (89.2%), permanent employees (94.5%), with a mean organizational tenure of 6.13 (*SD* = 6.53) and a mean age of 35.91 (*SD* = 10.54). Descriptive statistics, Pearson correlations, intercorrelation among latent constructs, skewness, and kurtosis are presented in Table 1.

We conducted several robustness checks. First, participants who completed all three waves were not significantly different from those who dropped out of Wave 2 in terms of doctoral incivility (*t* (361) = 0.750 and *p*=0.454), supervisor incivility (*t* (360) = 1.592 and *p*=0.112), coworker incivility (*t* (361) = 0.344 and *p*=0.731), and patient/visitor incivility (*t* (360) = −0.237 and *p*=0.812), and not significantly different from those who dropped out of Wave 3 in terms of doctoral incivility (*t* (402) = 0.482 and *p*=0.630), supervisor incivility (*t* (402) = 0.638 and *p*=0.524), coworker incivility (*t* (402) = 0.326 and *p*=0.744), and patient/visitor incivility (*t* (401) = 0.142 and *p*=0.887). These results suggested that missing data bias may not be a concern.

Second, we tested for potential gender differences in all study variables. The result indicated that there were no significant correlations between gender and any of the study variables (see Table 1), that is, there was no significant difference between women and men in all these study variables.

Finally, to use FIML, it requires continuous variables and normality. All the study variables (i.e., different sources of workplace incivility, the three basic psychological needs, and turnover intentions) were continuous variables. To ensure normality of the study variables, we examined their skewness and kurtosis. Statisticians (e.g., [50–52])) suggested that if the skewness (symmetry) range falls between −2 and +2 and the kurtosis (peakedness) range fails between −7 and +7, then the data distribution is considered normal. Except for the skewness of supervisor incivility, which was slightly above 2, the skewness and the kurtosis of all other study variables fell within the acceptable ranges, suggesting that these variables were normally distributed (e.g., [50–52]). Following previous research (e.g., [53, 54]), we conducted log-transformation for supervisor incivility and rerun our analyses. The results were the same with or without the log transformation. For the sake of easy interpretation, we kept the results without the log transformation of supervisor incivility. However, the results with log-transformation are available upon request.

### 3.2. Confirmatory Factor Analysis

Before testing our hypotheses, we conducted confirmatory factor analysis (CFA) to examine the distinctiveness of the study variables. Because our sample size was small relative to the number of items, we created three item parcels for each of the constructs with more than three items. We sequentially assigned items per parcel based on the highest to lowest item-to-construct loadings/correlations [55]. As shown in Table 2, the results supported the distinctiveness of the study variables because the hypothesized eight-factor model (*χ*^2^(224) = 350.60, *p* < 0.001, CFI = 0.98, RMSEA = 0.04, and SRMR = 0.04) outperformed all other alternative models, including the model with all incivility items loading onto a single factor (*χ*^2^(242) = 1606.01, *p* < 0.001, CFI = 0.74, RMSEA = 0.12, and SRMR = 0.08), and the model with all psychological need items loading onto a single factor (*χ*^2^(237) = 1351.43, *p* < 0.001, CFI = 0.79, RMSEA = 0.11, and SRMR = 0.09).

### 3.3. Hypotheses Testing

The results of the path analysis model without controls were mostly consistent with the results of the path analysis model with controls, except the path from patient incivility to the need for competence. Thus, following Becker's [56] recommendation, we reported the results with control variables (see Table 3). To examine our hypotheses, we compared two models: Model 1 with *free* estimates of the paths from each source of incivility to each psychological need and Model 2 with the paths from each source of incivility to each psychological need constrained to be equal. The comparison between Model 1 and Model 2 allowed us to test whether different sources of incivility would have differential relationships with the three psychological needs (hypotheses 2–4). The chi-square difference test indicated that the model with free estimates (a saturated model with perfect fit indices, *χ*^2^(0) = 0.00, *p* < 0.001, CFI = 1.00, RMSEA = 0.00 (90% CI (0.00 : 0.00)), and SRMR = 0.00) was better than the constrained model (*χ*^2^(9) = 27.38, *p* < 0.01, CFI = 0.95, RMSEA = 0.08 (90% CI (0.05 : 0.07)), and SRMR = 0.04).

**Figure 2:**
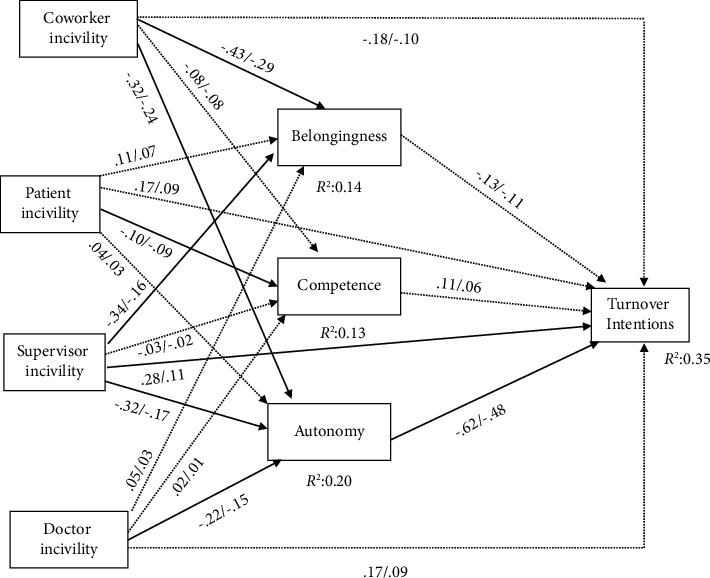
Relationships among workplace incivility, psychological needs, and turnover intentions. Note. Dash lines represent no significant paths, while solid lines represent significant paths. Values before the slash (/) are unstandardized coefficients, and values after the slash (/) are standardized coefficients.

We also tested Model 3 in which the paths from the three psychological needs to turnover intentions are constrained to be equal to compare with Model 1. The results indicated that Model 1 was significantly better than Model 3 (*χ*^2^(2) = 30.10, *p* < 0.001, CFI = 0.92, RMSEA = 0.21 (90% CI (0.15, 0.28)), and SRMR = 0.03). Therefore, the saturated model with the perfect model fit was retained as the final model. This model indicated that only supervisor incivility had a significant *direct* relationship with turnover intentions (*B* = 0.28, *SE* = 0.14, and *p* < 0.05), while other sources of incivility were not *directly* related to turnover intentions (see Table 2 and Figure 2).

Coworker incivility (indirect effect = 0.20, *SE* = 0.06, and *p* < 0.001), supervisor incivility (indirect effect = 0.20, *SE* = 0.08, and *p* < 0.01), and doctor incivility (indirect effect = 0.14, *SE* = 0.06, and *p* < 0.05) were negatively related to turnover intentions via reduced need for autonomy, thereby confirming hypothesis 1 regarding the mediating role of the psychological need for autonomy in the relationships of doctor incivility (H1a), supervisor incivility (H1b), and coworker incivility (H1c) with turnover intentions. However, no source of incivility influenced turnover intentions via psychological needs for belongingness or competence.

Doctor incivility was negatively related to the need for autonomy (*B* = −0.22, *SE* = 0.09, and *p* < 0.05) but not the need for belongingness (*B* = 0.05, *SE* = 0.11, and *p*=0.64) or the need for competence (*B* = 0.02, *SE* = 0.08, and *p*=0.85), supporting hypothesis 2a. Supervisor incivility was negatively related to the need for belongingness (*B* = −0.34, *SE* = 0.13, and *p* < 0.01) and the need for autonomy (*B* = −0.32, *SE* = 0.11, and *p* < 0.01) but not the need for competence (*B* = −0.03, *SE* = 0.09, and *p*=0.74). However, there was no significant difference in the coefficients for the relationships of supervisor incivility with the need for belongingness or the need for autonomy (difference = −0.02, *SE* = 0.13, and *p*=0.89), thus failing to support hypothesis 2b.

Coworker incivility was negatively related to the need for belongingness (*B* = −0.43, *SE* = 0.10, and *p* < 0.001) and the need for autonomy (*B* = −0.32, *SE* = 0.09, and *p* < 0.001), but not the need for competence (*B* = −0.08, *SE* = 0.07, and *p*=0.28). However, there was no significant difference in the coefficients for the relationships of coworker incivility with the need for belongingness or the need for autonomy (difference = −0.10, *SE* = 0.10, and *p*=0.31), thus failing to support hypothesis 3.

Patient incivility was not significantly related to the need for belongingness (*B* = 0.11, *SE* = 0.09, and *p*=0.22), the need for competence (*B* = −0.10, *SE* = 0.07, and *p*=0.13), or the need for autonomy (*B* = 0.04, *SE* = 0.08, and *p*=0.62), failing to support hypothesis 4.

Neither the need for belongingness (*B* = −0.13, *SE* = 0.07, and *p*=0.07) nor the need for competence (*B* = 0.11, *SE* = 0.09, and *p*=0.22) was related to turnover intentions. However, the need for autonomy was negatively related to turnover intentions (*B* = −0.62, *SE* = 0.08, and *p* < 0.001), supporting hypothesis 5.

## 4. Discussion

Our participants are New Zealand registered nurses, most of whom are women (consistent with the NZ nurse population [57]), permanent employees, with a mean organizational tenure of six years and a mean age of 36 years. Based on this sample, we have three main findings. First, when considering incivility from doctors, supervisors, fellow nurses, and patients/visitors, only supervisor incivility is directly related to nurse turnover intentions. Second, frustration of the autonomy need is the only significant mechanism explaining the relationships of incivility from doctors, direct supervisors, and fellow nurses with turnover intentions. Third, doctor incivility (i.e., top-down incivility) is related to the need for autonomy but not the need for belongingness nor the need for competence. Unexpectedly, the relationships of coworker incivility and supervisor incivility with the needs for belongingness and autonomy are equally significant, although neither coworker incivility nor supervisor incivility is significantly related to the need for competence. Finally, incivility from patients/visitors (i.e., outside incivility) is not significantly related to any psychological needs.

### 4.1. Theoretical Implications

Our findings have several theoretical contributions. First, our study sets to examine the association between incivility from four divergent sources and turnover intentions. In doing so, we distinguish whether and how incivility from doctors, direct supervisors, fellow nurses, and patients/visitors may be differently related to nurse turnover intentions, thereby responding to the calls for empirical investigations of incivility from multiple sources [17]. Consistent with previous research emphasizing the negative influence of supervisor incivility (e.g., [58]), it is the only one that is significantly, directly associated with turnover intentions. Due to their unique position, supervisors control important organizational resources such as pay allocation, promotion, and work assignments [59]. Thus, when considering all sources of experienced incivility, the only factor significantly related to turnover intentions is incivility from a supervisor. Therefore, we contribute to the incivility literature by highlighting the unique association between supervisor incivility and turnover intentions.

Second, we extend prior research that has taken a stress- and social exchange-perspective to understand the relationship between incivility and turnover intentions. Based on SDT [23], our study provides an alternative perspective to explain the incivility-turnover intentions association. We find that the need for autonomy is the only significant mechanism underlying the workplace incivility-turnover intentions relationship. Surprisingly, frustration of the psychological needs for belongingness and competence does not mediate the association between incivility and turnover intentions. However, this is consistent with the tenet of SDT that each basic psychological need can *independently* serve as an underlying mechanism explaining the relationships between work environments and outcomes [23]. Together, this result sheds light on the importance of the autonomy need in terms of nurses' intentions to stay or leave an organization.

Finally, our study is the first to provide evidence to support that incivility from different sources may be related to different basic psychological needs. Specifically, the results support the linkage between doctor incivility and the frustration of the autonomy need. Unanticipatedly, coworker and supervisor incivility are equally significantly related to the frustration of both belongingness and autonomy needs. Given that nursing tasks are primarily team-based and require interdependence among team members [60], it is possible that a nurse may not have the freedom to act with a sense of choice and volition when experiencing incivility from fellow nurses. As a result, the need for autonomy may be frustrated. It is also possible that nurse victims perceive their direct supervisor as an ingroup member, since the direct supervisor is essentially a nurse, such as the head of the nursing department. Moreover, according to the group value model [61], supervisor incivility signals that the victims are not valued group members [18]. Thus, incivility from one's direct supervisor also threatens the need for belongingness. In addition, patient/visitor incivility is not significantly related to any psychological need frustration. Since patients and visitors tend to have short-term relationships with nurses, it is plausible that incivility from patients and visitors is not a significant predictor of nurses' psychological needs. Our results echo the theorizing that incivility from different sources may trigger different victim reactions and highlight the importance of taking a nuanced perspective when examining workplace incivility. This novel investigation opens new opportunities to move from lumping together incivility from different sources to examining the role of specific instigators. In doing so, our findings add to a more comprehensive theory of workplace incivility by providing a fine-grained perspective of workplace incivility from multiple sources and its outcomes.

### 4.2. Practical Implications

In an ideal workplace environment, incivility would be reduced or eliminated entirely. However, assuming that the ideal cannot always be achieved, from a practical perspective, knowledge gained in this study has the potential to identify fruitful points of intervention to mitigate the impact of workplace incivility and break the chain of events that translate incivility into turnover intentions. Specifically, because supervisor incivility has a direct relationship with turnover intentions, it suggests that training to develop the leadership skills of nurse supervisors is needed. For example, Gonzalez-Morales et al. [62] have developed a short supervisor training to provide subordinates with supervisor support and reduce mistreatment from supervisors. Moreover, organizations may also implement transformational leadership training [63], authentic leadership training [64], or servant leadership training [65], all of which have been found to reduce employee turnover intentions. In addition, implementing a zero-tolerance policy for workplace incivility from all sources could also be effective in mitigating incivility [66].

Moreover, the empirical evidence suggests that the primary mediating mechanism explaining the relationship between incivility and turnover intentions concerns the frustration of the autonomy need. Thus, organizations should strive to ensure victims to have other means to fulfil their needs for autonomy. For example, derived from the job characteristics model [67], organizations may redesign jobs to facilitate task variety, task identity, task significance, job autonomy, and feedback [68]. Providing additional resources to victims (e.g., autonomy to freely choose their working hours and enhanced empowerment [69]) may not only boost basic need satisfaction but also indirectly reduce their turnover intentions. To maximize employees' basic need satisfaction, organizations may enable career development, offer opportunities to enhance employability, allow employees to participate in decision-making, offer performance feedback, and assign mentors to provide guidance, support, and knowledge [70].

### 4.3. Limitations and Future Research Directions

Although our study provides important insights, some limitations should be noted. First, the causality among variables of interest is only theory driven. Future research may use experimental designs to increase internal validity of our study conclusions.

Second, our study uses self-report data, which may suffer from common method bias. However, self-report data may be the best method for measuring variables of interest [71]. For example, what constitutes incivility is subjective, and perceptions of incivility may vary across individuals. Nonetheless, since incivility concerns the violation of mutual respect, gathering additional reports from multiple sources could provide a more accurate picture of the extent and nature of incivility in the workplace. To mitigate common method biases, we follow Podsakoff et al.'s [72] recommendation by separately measuring the predictors, the mediators, and the outcome with one workweek shift in between measurement points. Given that there is no agreement regarding the most appropriate reference period when measuring incivility [17], the chosen one-workweek shift as the time lag was primarily driven by occupational demands (cf. [73]). For example, working a 12-hour shift prevents nurses from participating in a daily diary study. On the other hand, nurses may not be able to accurately recall experienced incivility if a longer time lag (e.g., one month in between) was used [72].

To recruit participants, we used convenience sampling, a type of nonprobability or nonrandom sampling approach. Thus, the conclusions drawn from our study may be biased or impacted by outliers [74]. However, all variables of interest were within the normal ranges. We did not collect information about why some respondents dropped out during the study period. Nevertheless, we compared those who completed the study with those who dropped out and found no significant differences in any of the study variables. Hence, this may not be a significant concern.

The small sample size also necessitated conducting CFAs with parcels. Although creating parcels may have disadvantages such as masking cross-loadings at the item level [75]; in the current study, our aim was to understand the relationships among the latent constructs rather than among items. Therefore, conducting CFAs with parcels was recommended [76]. However, it is important to note that our hypotheses were not tested by SEM with parcels, but by path analyses without parcels. In addition, the sample was limited to nurses from New Zealand, which may affect the generalizability of our findings as the dynamics of patient care may differ in other countries.

Future research should examine the unique effects of multifoci incivility, such as its impact on the quality of patient care. In addition, studies could investigate the effectiveness of interventions mentioned earlier, such as supervisor training. Identifying potential moderators at the individual, workgroup, and organizational levels would provide insights on how to best intervene. Researchers could consider workgroup and organizational demands and resources (e.g., supervisor support and work overload) from a multilevel perspective, which may result in cross-level interactions either exacerbating or attenuating the relationship between workplace incivility and turnover intentions.

### 4.4. Conclusion

Although researchers believe that victims may react to incivility from different sources differently, research in this area of investigation is limited. Our research identifies that incivility from doctors is associated with the autonomy need, whereas incivility from patients/visitors is not associated with any psychological needs. Incivility from fellow nurses and supervisors is associated with the needs for belongingness and autonomy. Moreover, the psychological need for autonomy mediates the relationship of incivility from doctors, fellow nurses, and supervisors with turnover intentions. In doing so, our research takes a step toward addressing the important questions of how individuals may react differently to incivility from multiple sources.

## Figures and Tables

**Table 1:** Descriptive statistics, Pearson correlations, and intercorrelations among the latent constructs in the confirmatory factor analysis with parcels.

	1	2	3	4	5	6	7	8
(1) Coworker incivility T1	(0.90)	0.63	0.59	0.49	−0.39	−0.18	−0.48	0.30
(2) Supervisor incivility T1	0.56	(0.87)	0.48	0.33	−0.35	−0.09	−0.44	0.37
(3) Doctor incivility T1	0.53	0.42	(0.89)	0.48	−0.20	−0.17	−0.41	0.32
(4) Patient/visitor incivility T1	0.42	0.29	0.43	(0.87)	−0.09	−0.26	−0.27	0.22
(5) Need for belongingness T2	−0.32	−0.27	−0.15	−0.05	(0.85)	0.18	0.60	−0.41
(6) Need for competence T2	−0.15	−0.07	−0.14	−0.19	0.17	(0.89)	0.31	−0.13
(7) Need for autonomy T2	−0.39	−0.34	−0.32	−0.20	0.49	0.26	(0.81)	−0.64
(8) Turnover intentions T3	0.26	0.30	0.26	0.18	−0.34	−0.09	−0.55	(0.94)
(9) Gender	−0.01	−0.06	−0.02	0.03	0.08	−0.08	−0.02	−0.03
Mean	1.92	1.40	1.80	1.90	5.11	5.83	4.33	2.59
SD	0.79	0.58	0.72	0.75	1.15	0.84	1.04	1.36
Skewness statistics	0.86	2.34	1.09	0.69	−0.57	−0.85	−0.17	0.41
Skewness std. error	0.12	0.12	0.12	0.12	0.13	0.13	0.13	0.14
Kurtosis statistics	0.09	6.63	1.31	−0.08	−0.19	1.13	0.42	−1.10
Kurtosis std. error	0.24	0.24	0.24	0.24	0.27	0.27	0.027	0.29

*Note.* Cronbach's alpha is on the diagonal. Pearson correlations are below the diagonal. The intercorrelations among the latent constructs are above the diagonal. T1 = time 1; T2 = time 2; T3 = time 3. Wave 1: *N* = 413; wave 2: *N* = 339; wave 3: *N* = 294. Absolute values great than 0.14 in the Pearson correlations are significant at 0.05 level.

**Table 2:** Standardized factor loadings of the confirmatory factor analysis with parcels.

Parcels	FL
*Nurse incivility*	
Parcel 1	0.87
Parcel 2	0.80
Parcel 3	0.89

*Supervisor incivility*	
Parcel 1	0.92
Parcel 2	0.82
Parcel 3	0.72

*Doctor incivility*	
Parcel 1	0.86
Parcel 2	0.94
Parcel 3	0.80

*Patient incivility*	
Parcel 1	0.86
Parcel 2	0.79
Parcel 3	0.84

*Need for belongingness*	
Parcel 1	0.78
Parcel 2	0.87
Parcel 3	0.84

*Need for competence*	
Parcel 1	0.96
Parcel 2	0.89
Parcel 3	0.84

*Need for autonomy*	
Parcel 1	0.73
Parcel 2	0.84
Parcel 3	0.77

*Turnover intention*	
Parcel 1	0.91
Parcel 2	0.92
Parcel 3	0.92

*Note.* FL = factor loading. All factor loadings are *p* < 0.001.

**Table 3:** Parameter estimates of the two models.

Paths	Model 1		Model 2		Model 3		Model 4	
	B (SE)	(*β*) SE	B	(*β*) SE	B (SE)	(*β*) SE	B	(*β*) SE
Coworker incivility ⟶ belongingness	−0.43 (0.10)^*∗∗∗*^	−0.29 (0.07)^*∗∗∗*^	−0.16 (0.03)^*∗∗∗*^	−0.11 (0.02)^*∗∗∗*^	−0.43 (0.10)^*∗∗∗*^	−0.29 (0.07)^*∗∗∗*^	−0.42 (0.10)^*∗∗∗*^	−0.29 (0.07)^*∗∗∗*^
Patient incivility ⟶ belongingness	0.11 (0.09)	0.07 (0.06)	−0.16 (0.03)^*∗∗∗*^	−0.10 (0.02)^*∗∗∗*^	0.11 (0.09)	0.07 (0.06)	0.13 (0.09)	0.08 (0.06)
Supervisor incivility ⟶ belongingness	−0.34 (0.13)^*∗∗*^	−0.16 (0.06)^*∗∗∗*^	−0.16 (0.03)^*∗∗∗*^	−0.08 (0.01)^*∗∗∗*^	−0.34 (0.13)^*∗∗*^	−0.16 (0.06)^*∗∗∗*^	−0.31 (0.13)^*∗*^	−0.15 (0.06)^*∗*^
Doctor incivility ⟶ belongingness	0.05 (0.11)	0.03 (0.07)	−0.16 (0.03)^*∗∗∗*^	−0.10 (0.02)^*∗∗∗*^	0.05 (0.11)	0.03 (0.07)	0.03 (0.10)	0.02 (0.06)
Coworker incivility ⟶ competence	−0.08 (0.07)	−0.08 (0.07)	−0.05 (0.02)^*∗*^	−0.05 (0.02)^*∗*^	−0.08 (0.07)	−0.08 (0.07)	−0.07 (0.08)	−0.07 (0.07)
Patient incivility ⟶ competence	−0.10 (0.07)	−0.09 (0.06)	−0.05 (0.02)^*∗*^	−0.05 (0.02)^*∗*^	−0.10 (0.07)	−0.09 (0.06)	−0.20 (0.07)^*∗∗*^	−0.17 (0.06)^*∗∗*^
Supervisor incivility ⟶ competence	−0.03 (0.09)	−0.02 (0.06)	−0.05 (0.02)^*∗*^	−0.03 (0.01)^*∗*^	−0.03 (0.09)	−0.02 (0.06)	0.04 (0.10)	0.03 (0.06)
Doctor incivility ⟶ competence	0.02 (0.08)	0.01 (0.07)	−0.05 (0.02)^*∗*^	−0.04 (0.02)^*∗*^	0.02 (0.08)	0.01 (0.07)	−0.05 (0.08)	−0.04 (0.07)
Coworker incivility ⟶ autonomy	−0.32 (0.09)^*∗∗∗*^	−0.24 (0.07)^*∗∗∗*^	−0.21 (0.03)^*∗∗∗*^	−0.16 (0.02)^*∗∗∗*^	−0.32 (0.09)^*∗∗∗*^	−0.24 (0.07)^*∗∗∗*^	−0.31 (0.09)^*∗∗∗*^	−0.23 (0.06)^*∗∗∗*^
Patient incivility ⟶ autonomy	0.04 (0.08)	0.03 (0.06)	−0.21 (0.03)^*∗∗∗*^	−0.15 (0.02)^*∗∗∗*^	0.04 (0.08)	0.03 (0.06)	0.02 (0.08)	0.02 (0.06)
Supervisor incivility ⟶ autonomy	−0.32 (0.11)^*∗∗*^	−0.17 (0.06)^*∗∗*^	−0.21 (0.03)^*∗∗∗*^	−0.11 (0.01)^*∗∗∗*^	−0.32 (0.11)^*∗∗*^	−0.17 (0.06)^*∗∗*^	−0.31 (0.11)^*∗∗*^	−0.17 (0.06)^*∗∗*^
Doctor incivility ⟶ autonomy	−0.22 (0.09)^*∗*^	−0.15 (0.06)^*∗*^	−0.21 (0.03)^*∗∗∗*^	−0.14 (0.02)^*∗∗∗*^	−0.22 (0.09)^*∗*^	−0.15 (0.06)^*∗*^	−0.22 (0.09)^*∗*^	−0.15 (0.06)^*∗*^
Coworker incivility ⟶ TI	−0.18 (0.12)	−0.10 (0.07)	−0.18 (0.12)	−0.10 (0.07)	−0.13 (0.12)	−0.08 (0.07)	−0.15 (0.11)	−0.09 (0.07)
Patient incivility ⟶ TI	0.17 (0.10)	0.09 (0.06)	0.17 (0.10)	0.09 (0.05)	0.13 (0.11)	0.07 (0.06)	0.14 (0.10)	0.08 (0.06)
Supervisor incivility ⟶ TI	0.28 (0.14)^*∗*^	0.11 (0.06)^*∗*^	0.28 (0.14)^*∗*^	0.11 (0.06)^*∗*^	0.34 (0.15)^*∗*^	0.14 (0.06)^*∗*^	0.30 (0.14)^*∗*^	0.12 (0.06)^*∗*^
Doctor incivility ⟶ TI	0.17 (0.12)	0.09 (0.06)	0.17 (0.12)	0.09 (0.06)	0.26 (0.13)^*∗*^	0.13 (0.06)^*∗*^	0.16 (0.12)	0.08 (0.06)
Belongingness ⟶ TI	−0.13 (0.07)	−0.11 (0.06)	−0.13 (0.07)	−0.11 (0.06)	−0.26 (0.04)^*∗∗∗*^	−0.22 (0.03)^*∗∗∗*^	−0.13 (0.07)	−0.11 (0.06)
Competence ⟶ TI	0.11 (0.09)	0.06 (0.05)	0.11 (0.09)	0.06 (0.05)	−0.26 (0.04)^*∗∗∗*^	−0.15 (0.01)^*∗∗∗*^	0.12 (0.08)	0.08 (0.05)
Autonomy ⟶ TI	−0.62 (0.08)^*∗∗∗*^	−0.48 (0.06)^*∗∗∗*^	−0.62 (0.08)^*∗∗∗*^	−0.47 (0.06)^*∗∗∗*^	−0.26 (0.04)^*∗∗∗*^	−0.20 (0.03)^*∗∗∗*^	−0.62 (0.08)^*∗∗∗*^	−0.47 (0.06)^*∗∗∗*^

*Note.* TI = turnover intentions. Model 1: the model with the links from different sources to each psychological need and the links from psychological needs to turnover intentions were freely estimated. Model 2: the model with the links from different sources to each psychological need was constrained to be equal. Model 3: the model with the links from different psychological needs to turnover intentions was constrained to be equal. Model 4: this model is same as model 1 but without controls (sex: 1 = male, 2 = female, and 3 = gender diverse). We retained model 1 and discussed our findings based on model 1. ^*∗*^*p* < 0.05, ^*∗∗*^*p* < 0.01, and ^*∗∗∗*^*p* < 0.001.

## Data Availability

Anonymous data that support the findings of this study are available from the corresponding author upon reasonable request.
